# The feasibility of enhanced biological phosphorus removal in the novel oxic/extended idle process using fermentation liquid from sludge fermentation

**DOI:** 10.1039/c7ra12886j

**Published:** 2018-01-16

**Authors:** Yang Liu, Xiaoming Li, Jianwei Zhao, Dongbo Wang, Qi Yang, Guangming Zeng

**Affiliations:** College of Environmental Science and Engineering, Hunan University Changsha 410082 P. R. China zhaojianwei1213@yahoo.com xmli@hnu.edu.cn +86 731 88822829 +86 731 88823967; Key Laboratory of Environmental Biology and Pollution Control, Hunan University, Ministry of Education Changsha 410082 P. R. China

## Abstract

Carbon sources are essential for biological phosphorus removal (BPR); the carbon sources, however, are often inadequate in municipal wastewater treatment plants. This study demonstrated the feasibility of sludge fermentation liquid enhanced by biosurfactant alkylpolyglycosides (APG) as carbon sources to improve the performance of BPR in the novel oxic/extended idle (O/EI) reactor and the underlying mechanism was also investigated. The results showed that APG induced fermentation liquid could enhance the BPR performance in the O/EI reactor, and the BPR efficiency was 95.2%, which was significantly higher than that in the conventional anaerobic/oxic (A/O) reactor. Mechanism investigation showed that compared with the A/O reactor, the O/EI reactor enriched more polyphosphate accumulating organisms (PAOs) (38.2%), but less glycogen accumulating organisms (GAOs) when the APG-induced fermentation liquid was used as carbon source. The transformations of the polyhydroxyalkanoates (PHA) and glycogen in the O/EI reactor were lower than those in the A/O reactor. Further study found that the activities of polyphosphate kinase (PPK) and acetyl-CoA synthases (ACS) in the O/EI reactor were significantly higher than those of the A/O reactor, which was consistent with the higher BPR efficiency in the O/EI reactor.

## Abstract 2

Long-term effect of fermented liquid as carbon source on effluent COD and SOP in O/EI reactor.

## Graphical abstract

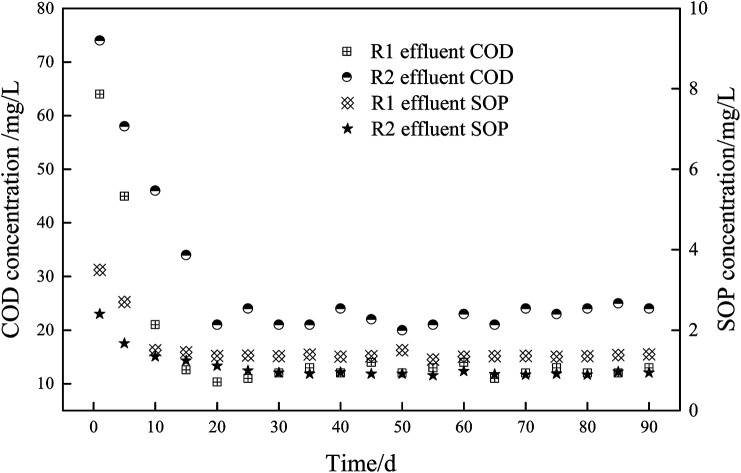

## 1. Introduction

Controlling phosphorus (P) emission is an important function of municipal wastewater treatment plants (WWTP) because P has been identified as the critical element responsible for eutrophication in the aquatic environment.^1^ Enhanced biological phosphorus removal (EBPR) is an economical and efficient method for phosphorus removal.^2^ EBPR mainly depends on the enrichment of polyphosphate accumulating organisms (PAOs) in the activated sludge to achieve phosphate removal.^3^ Under the anaerobic conditions, PAOs hydrolyze polyphosphates to gain energy to take up the available volatile fatty acids (VFAs) and store them in the form of internal carbon polymers, namely polyhydroxyalkanoates (PHAs). Under the subsequent oxic conditions, the PAOs degrade the PHAs stored anaerobically to generate energy for enhanced P uptake, maintenance metabolism and glycogen replenishment, and finally net P removal from the wastewater is achieved through the removal of waste activated sludge (WAS) containing a high level of polyphosphate.^4–6^ The EBPR efficiency, however, is often limited by insufficient carbon sources in influent especially in southern China. Adding external carbon source is a commonly used strategy to improve the efficiency of EBPR. However, external carbon sources are usually expensive and increase the operating burden of the WWTPs.^7,8^ Therefore, the development of economical and efficient carbon source is a hotspot of research.

Anaerobic fermentation of WAS is considered as a commonly used method to treat sludge. Recently, the carbon-rich fermentation liquid serving as an alternative preferred carbon source for EBPR and denitrification was widely studied.^9,10^ Ji and Chen^9^ found that using sludge fermentation liquid as carbon source can facilitate short-range nitrification and denitrification, and the removal efficiencies of nitrogen and phosphorus were higher than those using common carbon sources. Gao *et al.*^11^ proposed an innovative mode of operation (a 2-step sludge alkaline fermentation process and an anaerobic-anoxic-oxic (A^2^/O) reactor) to utilize sludge fermentation liquid to enhance nutrient removal. In addition, the utilization of sludge alkaline anaerobic fermentation liquid can reduce the production of nitrous oxide, a greenhouse gas.^12^ Although a large amount of studies have been conducted on the utilization of fermentation liquid to enhance biological phosphorus removal, previous studies have mostly focused on the traditional wastewater phosphorus removal process (such as anaerobic/oxic (A/O), A^2^/O) and most of the fermentation liquid originated from alkaline fermentation.^11^ It is well known that different fermentation types produce different components of the fermentation liquid, however, the potential impact of fermentation liquid from other fermentation methods on the performance of EBPR induced by innovative P removal process is rarely investigated.

Recently, EBPR can be achieved in a new mode *i.e.*, the anaerobic process is removed but the idle period is extended appropriately such as 210 min.^13,14^ The novel BPR mode, defined as oxic/extended idle regime (O/EI), has the features of simple operating procedure, stable and efficient phosphorus removal, and low dependence on carbon sources.^15^ Further research confirmed that the enrichment mode and metabolic pathway of PAOs in the O/EI regime were significant different from those in the traditional A/O process (*e.g.*, a significant idle release of phosphate and a low idle production of PHA).^16,17^ Therefore, the new EBPR technology has broad application prospects.

Biosurfactants are commonly used in sludge anaerobic fermentation because they can accelerate the sludge hydrolysis, acidification but inhibit methanogenesis thereby leading to the accumulation of volatile fatty acids (VFA). Among those biosurfactants, alkyl polyglycoside (APG) has attracted much attention due to its advantages of non-toxicity, easy degradation, and high sludge treatment efficiency.^18,19^ It was reported that the level of VFA from sludge fermentation can be as high as 282.9 mg COD per g VSS, and the content of acetic acid ranked highest when the dosage of APG was 0.2 g g^−1^ dry sludge (DS). Because most of the reagents used in the alkaline fermentation are NaOH, Na^+^ will be inevitably doped in the fermentation liquid, which will reduce the potential for subsequent utilization of C-rich fermentation liquid. However, APG is easily biodegradable so that no additional impurities are introduced into the fermentation liquid by the addition of reagents. Therefore, it is conceivable that APG-induced carbon-rich fermentation liquid may have more potential for subsequent bioavailability. However, there are few studies on the APG-induced fermentation liquid' utilization, especially for BPR.

Therefore, the purpose of this study is to first explore the feasibility of APG-induced fermentation liquid for the novel O/EI EBPR process. Secondly, the performances of between the novel O/EI regime and conventional A/O regime using the APG-induced fermentation liquid as carbon source were systematically compared. Finally, the mechanisms that O/EI process exhibiting better phosphorus removal using APG-induced fermentation liquid is deeply investigated. This study provides a strategy for reducing the production of sludge and improving the EBPR efficiency by providing APG-induced fermentation liquid as carbon source in the novel O/EI process.

## 2. Materials and methods

### 2.1 Sludge fermentation liquid preparation

The concentrated WAS used in this experiment was taken from the sludge pipeline of the secondary sedimentation tank of a full-scale municipal wastewater treatment in Changsha, China. The biosurfactant APG used in this study was purchased from Nanjing Duly Biotech Co. Ltd. (Jiangsu Province, China) and placed in a refrigerator at 4 °C for further use. The main characteristics of APG are as follows: solid content 50%, density 1.10 g cm^−3^. The detailed sludge fermentation conditions are presented in the ref. 17. The release of large amounts of phosphate into the fermentation liquid was found (ammonium release was also found, but the study focused on phosphates), so phosphate removal from the fermentation liquid was required to reduce its load. Phosphate in the fermentation liquid was removed by struvite crystallization method, the specific operating procedures were described in the literature.^20^ The main characteristics of the fermentation liquid after phosphate removal are shown in Table 1.

**Table 1:** The main characteristics of influent and fermentation liquid used in this experiment^a^

	Synthetic wastewater	Fermentation liquid	Influent
pH	6.8 ± 0.1	5.9 ± 0.1	6.5 ± 0.1
COD/mg L^−1^	150 ± 8	2105 ± 189	279 ± 14
VFA/mg L^−1^	189 ± 11	3415 ± 141	388 ± 15
NH_4_^+^-N/mg L^−1^	30 ± 3	285 ± 12	49 ± 3
SOP/mg L^−1^	8 ± 0.3	8.7 ± 0.4	10 ± 0.4

a Results are the averages and their standard deviations.

### 2.2 Synthetic wastewater and inoculation sludge

Synthetic wastewater was used in this experiment, and the synthetic wastewater contained sodium acetate, NH_4_Cl, KH_2_PO_4_, MgSO_4_, and CaCl_2_. The initial concentrations of COD, soluble phosphate (SOP), and NH_4_^+^-N were controlled at 150.0, 8.0 and 30.0 mg L^−1^, respectively. In addition, 0.5 mL L^−1^ of a trace element solution is also added to enhance the activity of microorganisms.^21^ The main characteristics of the synthetic wastewater are also presented in Table 1.

Inoculated sludge was taken from the biological reactor of Changsha municipal wastewater treatment plant. The inoculated sludge has good biological activity. The main properties of inoculated sludge are as follows: total suspended solids (TSS) 8000 mg L^−1^, volatile suspended solids (VSS) 4500 mg L^−1^, pH 6.7.

### 2.3 The feasibility of fermentation liquid to enhance BPR in the innovative O/EI reactor

Two identical reactors with working volume of 2.0 L were established and the operating procedures of two reactors were as follows: 210 min oxic period, followed by 60 min settling and decanting, and 210 min idle period according to our previous literature.^15^ The reactor was made of plexiglas and equipped with a stirrer, and the speed was controlled at 120 rpm. Temperature controlled at 30 °C in a room with air conditioning. The two reactors first received 1000 mL of inoculated sludge and 1000 mL of influent water. The influent water in one reactor (R1) was a mixture of synthetic wastewater and fermentation liquid (25/1 by volume). In contrast, the influent water in the other reactor (R2) was only the same volume of synthetic wastewater. The initial pH of the influent was adjusted to 7.0 by 2.0 M HCl or NaOH and the pH was not controlled during the entire process. By comparing the effluent characteristics of the two reactors, it was possible to determine the feasibility of the fermented liquid as carbon source to improve BPR in the novel O/EI process.

### 2.4 Comparison of BPR performances between the A/O and O/EI reactors using fermentation liquid

Two identical sequencing batch reactors (SBR) with working volume of 2.0 L were set up. One reactor was defined as A/O reactor and the other reactor was defined as OE/I reactor. The A/O and OE/I reactors operated three cycles daily, each containing eight hours. The OE/I reactor's operating procedure is described in Section 2.2. The A/O reactor's operating procedure consisted of a 120 min anaerobic period, followed by a 180 min oxic period, and 60 min settling and decanting period, and 120 min idle phases according to the literature.^20,22^ Other reaction conditions are described in Section 2.2. Both reactors were operated continuously for 90 days, and a comparison of BPR performance during a typical period was implemented after both reactors were stable.

### 2.5 Analytical method

The determinations of SOP, NH_4_^+^-N, COD, TSS and VSS were based on the literature.^17,23–26^ VFA was analyzed by Ailgent 6890 GC with flame ionization detector, the detailed measurement method was as follows: the samples were centrifuged at 5000*g* for 20 min before the determination, and the supernatant was diluted appropriately multiple times to make the concentration of VFA less than 200 mg L^−1^, then filtered with 0.45 μm filter membrane to remove particulate matter. The filtrate was collected in 1.5 mL gas chromatography special bottle, then added 50–150 μL H_3_PO_4_, so that pH value of sample was less than 3. The gas chromatographic conditions were in the literature.^24^ The determination of poly-3-hydroxybutyrate (PHB), poly-3-hydroxyvalerate (PHV), and poly-3-hydroxy-2-methylvalerate (PH2MV) was detected using gas chromatography, PHA was the sum of PHB, PHV, and PH2MV, the detailed determination was mainly as follows: (1) immediately add formaldehyde to the sludge to inhibit microbial metabolism (formaldehyde concentration of 1%). (2) After weighing and freeze-drying, 2 mL chloroform, 2 mL sulfuric acid methanol, and 0.2 mL benzoic acid methanol were added into 100 mg sludge sample to eliminate 20 h at 105 °C. (3) The samples were cooled and mixed with 1 mL distilled water, and then centrifuged 5000*g* for 15 min, the organic phase in the lower layer was analyzed by gas chromatography, other determination methods were detailed in the literature.^27–30^ The analyses of exopolyphosphatase (PPX) and polyphosphate kinase (PPK) and ACS, and microbial community were detailed in the literature.^17^

### 2.6 Statistical analysis

All experiments were conducted in triplicate. An analysis of variance (ANOVA) was employed to assess the significance of the results, and *p* < 0.05 was recognized to be statistically significant.

## 3. Results and discussion

### 3.1 Feasibility of fermentation liquid as carbon source to improve BPR in the O/EI regime

**Fig. 1:**
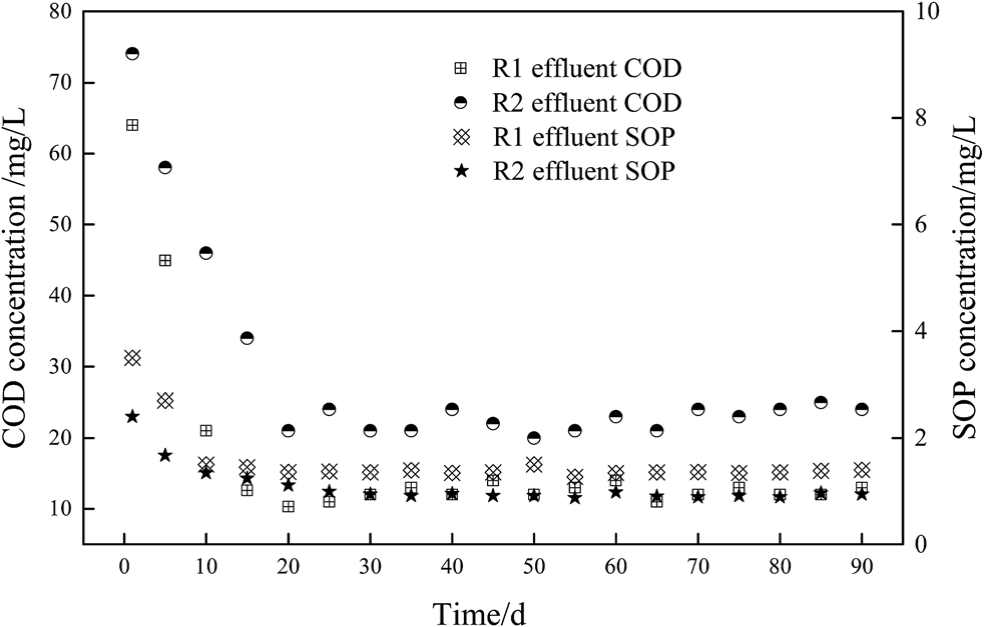
Long-term effect of fermented liquid as carbon source on effluent COD and SOP in O/EI reactor.

The presence of sufficient VFA in wastewater is a prerequisite to achieve excellent BPR,^31,32^ the effect of fermentation liquid addition on the operating performance of the novel O/EI reactor was shown in Fig. 1. As shown in Fig. 1, it took around 20 days for the effluent COD and SOP of both reactors to reach a steady state. During the stable operation, the effluent COD and SOP concentrations in the R1 reactor were 12.5 and 1.36 mg L^−1^, respectively, implying the removal efficiencies of COD and SOP were 93.6 and 83%, respectively. In this study, the COD removal rate was similar to previous studies,^14,33^ the SOP removal efficiency, however, was significantly lower than previous studies (*i.e.*, 83% *vs.* 95.7%), which may be attributed to the low concentration of influent COD in this study. When the APG-induced fermentation liquid was applied to the O/EI reactor, the level of effluent COD increased to 23 mg L^−1^, the removal rate of COD was 94.1%. APG-induced fermentation liquid contains variety of VFAs, this VFA-rich liquid can be used as preferred carbon source for enhancing microbial activity, including PAOs. The enhanced microbial activity is beneficial for increasing the consumption of influent organic matter, resulting in enhanced COD removal efficiency. The concentration of effluent SOP in R2 decreased to 0.89 mg L^−1^ and the corresponding removal efficiency of SOP was 91.1%, which was significantly higher than that in R1. The addition of APG-induced fermentation liquid not only increased the influent VFA content but also increased the organic load and nutrient load. The increase of nutrient load in this study had no obvious effect on the subsequent EBPR. The above experimental results clearly showed that the APG-induced fermentation liquid significantly improved the BPR efficiency in the novel O/EI reactor, suggesting APG induced fermentation liquid can be used as carbon source to enhance BRR in the novel O/EI reactor.

### 3.2 Comparison of BPR in the A/O and O/EI reactors using APG induced fermentation liquid

The phosphorus removal mechanism of the O/EI reactor is significantly different from that in the conventional A/O process.^15^ When the fermentation liquid is used as carbon source for phosphorus removal, the difference of EBPR performance is not clear. Therefore, it is necessary to explore the effect of APG induced fermented liquid as carbon source on EBPR performance of two reactors. Table 2 summarized the EBPR performances between the conventional A/O and the novel O/EI reactors during the stable operation period. It can be seen that both reactors had satisfactory COD and NH_4_^+^-N removal. However, high concentrations of nitrates in both reactors were found in the effluent because there was no strict anoxic phase in both reactors and the denitrification process was limited. In the A/O and O/EI reactors, total nitrogen remove efficiencies were 57.6% and 59.3%, respectively. In general, nitrogen in sewage is mainly removed through microbial assimilation and denitrification processes. Because of the insignificant variation in VSS for both reactors (data not shown), it can be concluded that aerobic denitrification occurred in both reactors due to the long oxic period in both reactors, which was consistent with our previous study.^14^

**Table 2:** Comparison of the reactor performances between A/O and O/EI using APG-induced fermentation liquid during the stable operation^a^

Item	A/O reactor	O/EI reactor
Effluent pH	7.8 ± 0.1	8.1 ± 0.2
COD removal efficiency/%	93.8	94.1
Effluent SOP/mg L^−1^	1.8 ± 0.2	0.89 ± 0.08
SOP removal efficiency/%	82 ± 4	91 ± 5
Effluent NH_4_^+^-N/mg L^−1^	2.84 ± 0.12	2.79 ± 0.1
Effluent NO_2_^−^-N/mg L^−1^	0.13 ± 0.03	0.15 ± 0.04
Effluent NO_3_^−^-N/mg L^−1^	13.52 ± 0.61	12.85 ± 0.56

a Results are the averages and their standard deviations.

As for the SOP removal, the two reactors showed obvious differences. The stable concentration of effluent SOP in the A/O reactor was about 1.8 mg L^−1^ and the corresponding phosphorus removal efficiency was around 82%. However, in the O/EI reactor, the effluent concentration of SOP decreased to 0.89 mg L^−1^, accompanied by phosphorus removal efficiency of 91%, indicating that compared with the traditional A/O reactor, the application of APG-induced fermentation liquid can lead to a higher phosphorus removal performance in O/EI reactor. The potential mechanism by which the O/EI reactor induced higher phosphorus removal efficiencies will be explored in detail below.

### 3.3 Mechanism for the O/EI reactor driving higher BPR performance using APG induced fermentation liquid

**Fig. 2:**
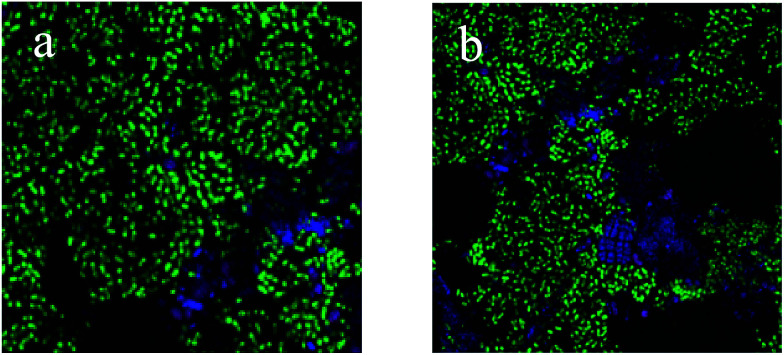
FISH micrographs of microbial communities from the O/EI (a) and A/O (b) reactors. Hybridizing with PAOmix (blue), GAOmix (red) and EUBmix (green) probes specific for *Accumulibacter* (PAOs), *Competibacter* and *Defluviicoccus*-related organisms (GAOs) and the dominant bacteria, respectively. Cells that were yellow had hybridized with both GAOmix and EUBmix probes. Samples were obtained after stable operation.

PAOs are the main microorganisms responsible for the removal of phosphate from wastewater, and the relative abundance of PAOs in activated sludge can directly reflect the BPR performance.^34^ As shown in Fig. 2, the relative abundance of PAOs in the O/EI reactor was 38.2%, significantly higher than that in A/O reactor. In addition, the relative abundance of GAOs in the O/EI reactor was lower than that of the A/O reactor. The metabolic activities of GAOs are similar to those of PAOs, but GAOs do not contribute to BPR, and excessive proliferation of GAOs competes with the PAOs for limited available carbon sources in the wastewater, resulting in a decrease in phosphorus removal efficiency.^15^ The high relative abundance of PAOs but low relative abundance of GAOs in the O/EI reactor was the main reason for its high BPR efficiency.

**Fig. 3:**
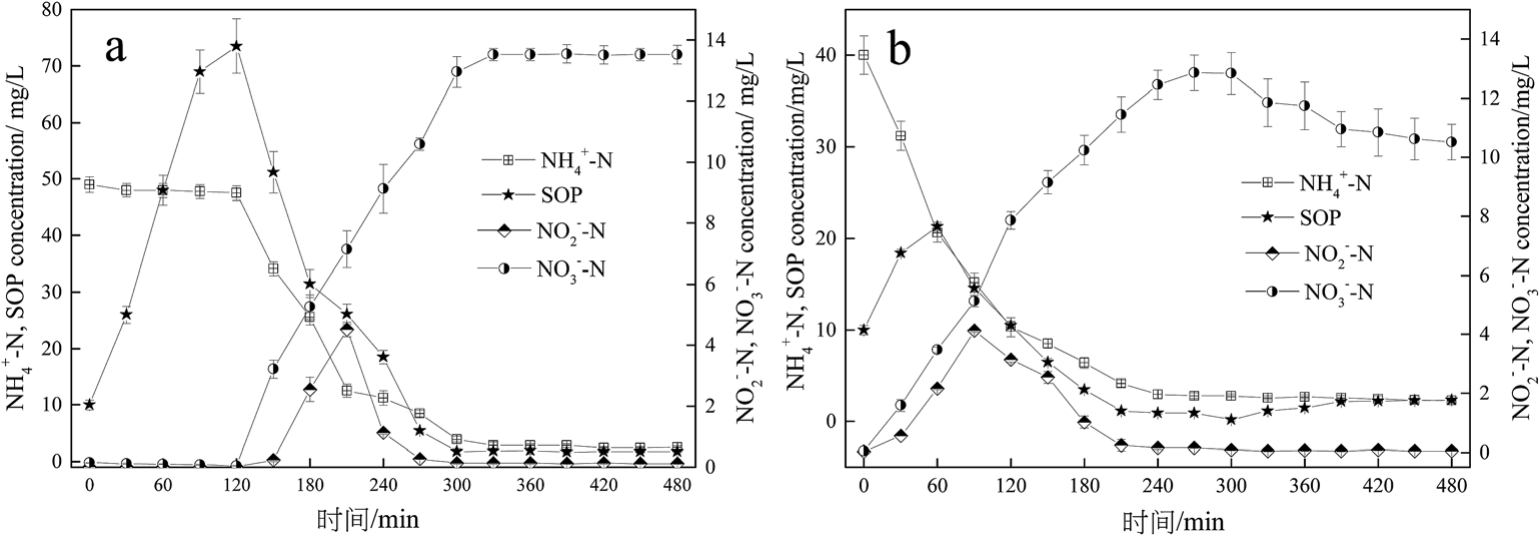
Comparison of nutrient changes in a typical cycle between the A/O reactor (a) and O/EI reactor (b). Results are the averages and their standard deviations of three different measurements.

The typical profiles of nutrients change in the A/O and O/EI reactors were shown in Fig. 3. In the A/O reactor, the concentration of NH_4_^+^-N decreased slightly which mainly due to the assimilation of ammonia nitrogen by microorganisms during the anaerobic period. During the subsequent oxic period, NH_4_^+^-N was quickly oxidized into NO_2_^−^-N and NO_3_^−^-N, resulting in a sharp decline of the NH_4_^+^-N concentration, and the effluent NH_4_^+^-N concentration was 2.84 mg L^−1^. The concentrations of NO_2_^−^-N and NO_3_^−^-N were almost zero during anaerobic phase but rose rapidly during aerobic phase with the maximum concentration of NO_2_^−^-N and NO_3_^−^-N being 4.51 and 13.52 mg L^−1^, respectively. The SOP concentration increased sharply to 73.5 mg L^−1^ during the anaerobic phase, and during the subsequent oxic phase, the SOP was uptaked luxuriously by the PAOs in the activated sludge, resulting in a rapid decline in the SOP concentration. At the end of the aerobic phase, the SOP concentration dropped to 1.90 mg L^−1^. The above nutrient changes in typical cycle were consistent with those in previous studies.^33,35^ In the O/EI reactor, the concentration of NH_4_^+^-N showed a downward trend over time, the content of NH_4_^+^-N in the effluent was only 2.84 mg L^−1^, suggesting that NH_4_^+^-N removal rate of up to 92.9%. NO_2_^−^-N increased first and then decreased during the oxic period, which was mainly related to the oxidation of NH_4_^+^-N and NO_2_^−^-N, and the maximum concentration of NO_2_^−^-N was 4.12 mg L^−1^, which was lower than the maximum accumulation of NO_2_^−^-N in the A/O reactor. NO_2_^−^-N, especially its protonated form free nitrite acid (FNA), had a strong inhibitory effect on the activity of PAOs.^36–38^ FNA can seriously inhibit the uptake of phosphate by PAOs.^35^ Therefore, the low concentration of nitrite accumulation in the O/EI reactor was one of the reasons for its high BPR efficiency. The concentration of NO_3_^−^-N increased continuously in oxic phase with the NO_3_^−^-N concentration in the effluent being as high as 12.85 mg L^−1^, which was similar to that in the A/O reactor. A large amount of nitrate accumulated in the supernatant would enter the next operating cycle. According to previous studies, O/EI regime can show better phosphorus removal performance under the same nitrate loading conditions than the A/O reactor.^14,15^ As for the changes of SOP, the SOP was released in the initial stage of aerobic. During the late aerobic period, the SOP was luxury uptake and the effluent SOP content was only 0.89 mg L^−1^ which was significantly lower than that of AO reactor. The reason why the O/EI reactor showed better BPR may be attributed to the metabolism mode of PAOs in the O/EI reaction system.

**Fig. 4:**
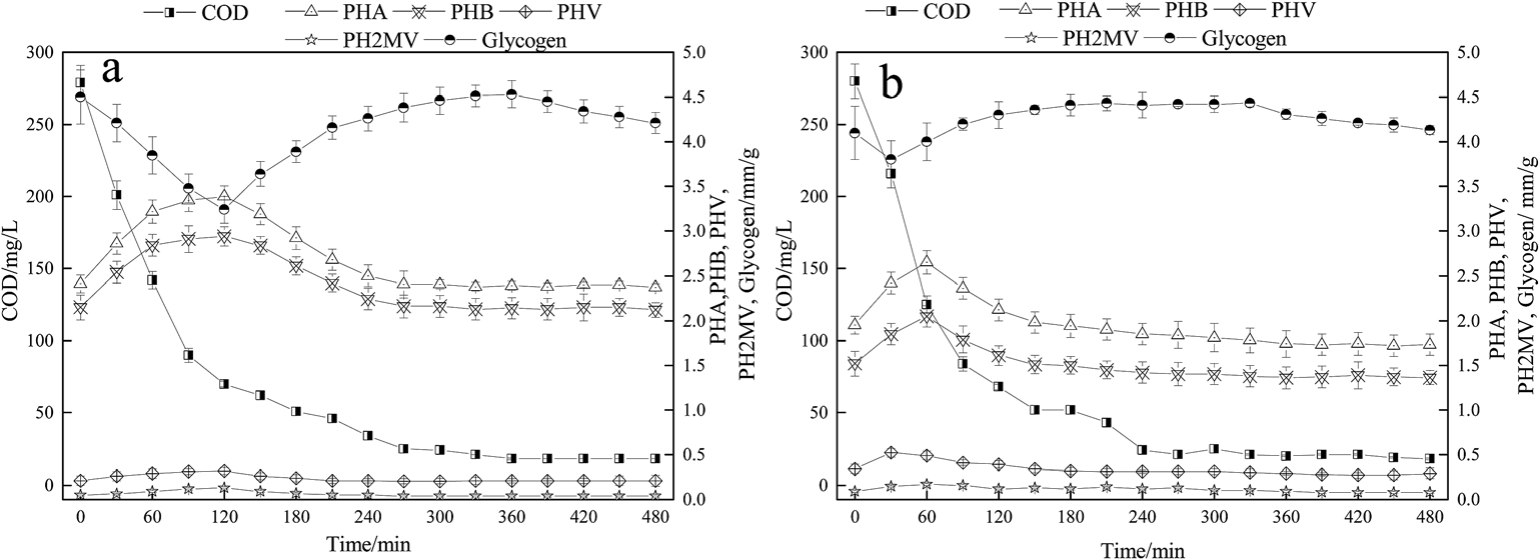
Comparison of COD and intracellular polymers changes in a typical cycle between the A/O reactor (a) and O/EI reactor (b). Results are the averages and their standard deviations of three different measurements.

The changes of intracellular polymers are closely related to the energy metabolism of microorganisms.^4^ Currently, the traditional BPR theory concludes that PAO absorbs VFA during the anaerobic period and stores them in the form of intracellular PHA. At the same time, the glycogen is degraded to produce reducing power. During the aerobic phase, PHA oxidizes to produce energy for over-uptake of phosphate. The content of PHA is closely related to the oxidation capacity, which affects the uptake of phosphate. Fig. 4 showed the variations in COD, and the intracellular polymers PHA and glycogen. In the A/O reactor, COD was quickly consumed within the first 240 min accompanied by the synthesis of PHA, and the maximum amount of PHA synthesized was 3.39 mm g^−1^ (calculated as mm-C in per g VSS) at the end of the anaerobic period. In the A/O reactor, the content of glycogen decreased from 4.50 mm g^−1^ at the beginning stage of anaerobic period to 3.24 mm g^−1^ in the late stage of anaerobic period, which was mainly due to the reduction of glycogen to produce reducing power for the synthesis of PHA. During the oxic phase, the content of glycogen gradually increased from 3.24 mm g^−1^ to 4.51 mm g^−1^, which is related to the degradation of PHA to produce energy for replenishing glycogen. At the end of oxic phase, the content of glycogen is similar to that of the initial stage of anaerobic phase. In the O/EI reactor, the COD content was also rapidly consumed, which was similar to that in the A/O reactor. As for PHA and the glycogen in the O/EI reactor, however, the changes of PHA and glycogen showed great differences from those in the A/O reactor. In the O/EI reactor, the content of PHA increased in the first 60 min and then rapidly degraded. Similarly, the content of glycogen degraded in the first 60 min and then gradually increased. According to the traditional metabolism theory of EBPR, the energy generated by the degradation of PHA during oxic phase is used to maintain its metabolism, supply the glycogen, and uptake the phosphorus.^4,39,40^ The amount of metabolisms in the two reactors were about the same (in terms of VSS, data not shown) whereas in the A/O reactor, the supply of glycogen was much higher than the O/EI reactor (0.82 mm g^−1^*vs.* 0.46 mm g^−1^). The amount of energy produced by the degradation of PHA for glycogen replenishment in the A/O reactor was much greater than in the O/EI reactor, suggesting more energy from the degradation of PHA was used for oxic phosphorus uptake in the O/EI reactor. Less energy used for excessive phosphorus uptake is also one of the reasons leading to low BPR efficiency in A/O reactor.

It is well-known that the efficiency of EBPR is closely associated with the change of the intracellular polymers PHA and glycogen in one typical cycle. In the A/O reactor, the PHA and glycogen changes were significantly higher than those in the O/EI reactor. It was reported that the variations of PHA and glycogen are related to the activities of PAOs and GAOs, and higher glycogen metabolic transformation implied higher GAOs activity.^15^ Therefore, lower glycogen metabolic transformation detected in O/EI reactor indicated lower activity of GAOs, which was consistent with the results in Table 2. In addition, the idle period in the O/EI reactor was much longer than that of the A/O reactor (210 min *vs.* 120 min). Phosphate release occurred during the OEI idle period with a net phosphorus release of 1.42 mg L^−1^, 4.7 times that of the A/O reactor. PHA, glycogen, and polyphosphate are the three main intracellular polymers. Under oxic conditions, PHA is easy to decompose and polyphosphate is difficult to degrade, however, polyphosphate can be easily degraded in anaerobic environment.^12,41^ In the A/O reactor, the energy required for the metabolism during the idle phase mainly came from the degradation of the glycogen. However, during the idle period of the O/EI reactor, the contents of PHA and glycogen returned to their original contents, therefore, the energy required for the metabolism of microorganisms mainly came from the degradation of polyphosphorus, which enhanced the action of PAOs in the activated sludge. It has been reported that the function of polyphosphorus relates to the activity of PAO, and the greater the role of polyphosphorus in the metabolic transformation of microorganisms, the stronger the activity of PAO.^4,15^

### 3.4 Effect of fermentation liquid as carbon source on key enzymes responsible for BPR

**Fig. 5:**
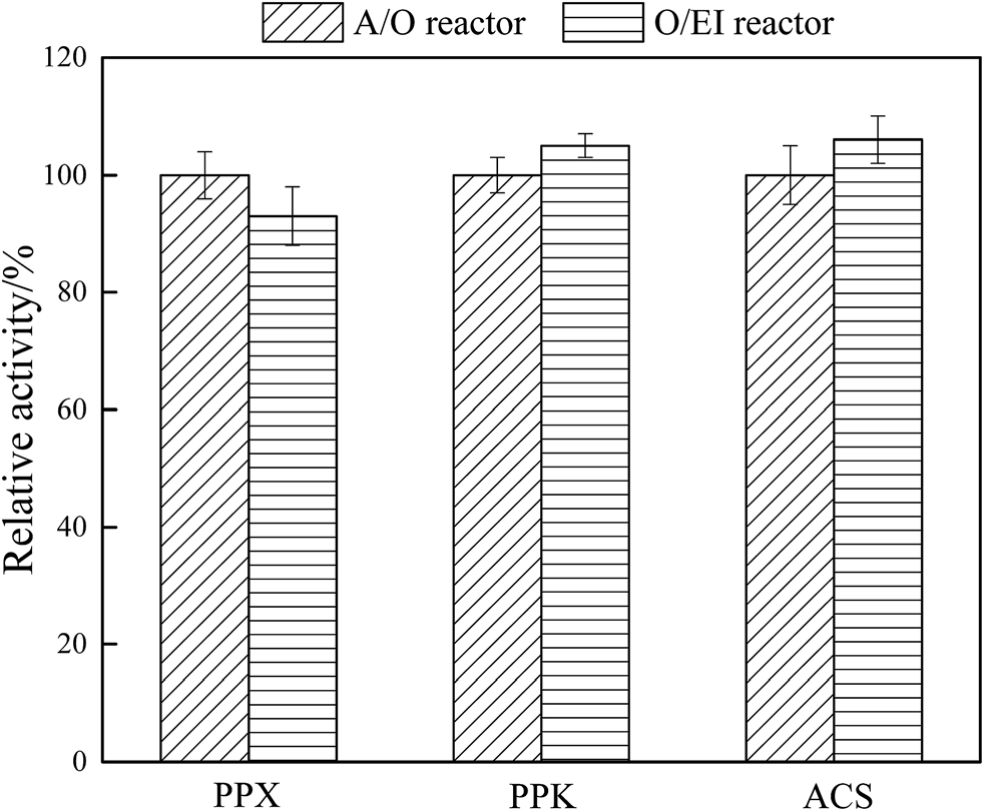
Comparison of the activities of key enzymes responsible for EBPR during the stable operation. The activities of the key enzymes in the A/O reactor was set as 100%. Results are the averages and their standard deviations of three different measurements.

Exploring the activities of key enzymes is an important way to understand microbial metabolism. PPX, PPK, and ACS are the key enzymes responsible for BPR, and PPX and PPK are closely related to anaerobic phosphorus release and oxic phosphorus uptake.^2^ ACS mainly catalyzed the conversion of acetic acid to PHAs precursor acetyl coenzyme A.^42^ The effect of APG-induced fermentation liquid as carbon source on the key enzyme activities in stable operation of A/O and O/EI reactors is shown in Fig. 5. The activity of PPX in the O/EI reactor was lower than that in the A/O reactor, which was consistent with less SOP release in the O/EI reactor. The special operating condition *i.e.* canceling the anaerobic phase, in the O/EI reactor leads to the decrease of PPX activity in activated sludge. The activities of PPK and ACS in the O/EI reactor were significantly higher than those in the A/O reactor (*p* < 0.05), which was also consistent with the higher efficiency of BPR in the O/EI reactor. Polyphosphate metabolism is the key metabolic program for PAOs, the activity of PPK is closely related to aerobic phosphorus uptake and synthesis of polyphosphate. The higher relative PPK activity detected in the O/EI reactor is also an important reason for the O/EI regime exhibiting higher BPR performance.

## 4. Conclusion

The feasibility of using APG-induced sludge fermentation liquid for the novel O/EI reactor to enhance BPR was demonstrated in this study. When APG-induced fermentation liquid was used as the carbon source, the removal efficiency SOP in the OEI reactor was 95.2%, which was higher than that in the A/O reactor. The mechanism study showed that the O/EI reactor enriched more PAOs but less GAOs, and higher activities of PPK and ACS, lower transformation of glycogen, which were the main reasons for the higher BPR performance achieved in the O/EI reactor.

## Conflicts of interest

There are no conflicts to declare.

## Supplementary Material
